# Determining the Importance of Macro and Trace Dietary Minerals on Growth and Nutrient Retention in Juvenile *Penaeus monodon*

**DOI:** 10.3390/ani10112086

**Published:** 2020-11-10

**Authors:** Ha H. Truong, Amy F. Moss, Nicholas A. Bourne, Cedric J. Simon

**Affiliations:** 1 Bribie Island Research Centre, CSIRO Agriculture and Food, Woorim 4507, Australia; 2 School of Environmental and Rural Science, University of New England, Armidale, NSW 2351, Australia; amoss22@une.edu.au; 3 Queensland Bioscience Precinct, CSIRO Agriculture and Food, Brisbane 4067, Australia; nicholas.bourne@csiro.au (N.A.B.); Cedric.Simon@csiro.au (C.J.S.)

**Keywords:** crustacean aquaculture, shrimp, mineral requirements, body composition, nutrient retention

## Abstract

**Simple Summary:**

Knowledge of mineral requirements enables diets to be better formulated. Mineral requirements of black tiger prawns (*Penaeus monodon*) are not well known compared with other cultured prawn species. To close this knowledge gap, the importance of providing additional sources of twelve minerals in prawn diets were assessed. These minerals are known to be required for optimal growth in other animals and included boron, calcium:phosphorus at 1:1 ratio, cobalt, copper, magnesium, manganese, potassium, selenium, sodium, strontium and zinc. Inorganic forms of these minerals were incorporated into diets and fed to prawns for 6 weeks where their effect on growth performance and mineral concentrations in tissues were determined. This study was able to assess the effect of many minerals by adopting a ‘screening design’ where it was demonstrated that additions of calcium:phosphorus at 1:1 ratio, magnesium, boron, manganese, selenium and zinc to diets for black tiger prawns were important for growth, feed conversion efficiency and nutrient utilisation. Further research is needed to determine the requirement values of the important minerals identified in this study.

**Abstract:**

Twelve minerals were screened to identify key dietary minerals important for *Penaeus monodon* growth. The minerals selected included boron, calcium plus phosphorus (assessed in tandem at a 1:1 ratio), cobalt, copper, magnesium, manganese, potassium, selenium, sodium, strontium and zinc. Twelve purified casein/gelatin-based diets were formulated and contained minerals at two levels: below expected requirements, as attributed by the basal formulation (−) and above expected requirements by adding inorganic minerals (+). The two levels were allocated to dietary treatments in juvenile prawns in accordance with the PB design. A two-level screening analysis was employed where effect of each mineral at level − or + across twelve diets were compared to identify the minerals of importance for culture performance of juvenile prawns. Calcium plus phosphorus (at a 1:1 ratio), magnesium, boron, manganese, selenium and zinc produced the greatest positive effects on weight gain, feed conversion efficiency, biomass gain and nutrient/energy retention. Particularly, boron and manganese significantly increased retention of key macronutrients and energy including gross energy, crude protein and crude lipid. Our study demonstrates the importance of several macro and trace minerals in prawn diets and the pressing need to refine their requirements for *P. monodon*.

## 1. Introduction

Dietary minerals are needed for a myriad of biological processes. For terrestrial livestock, requirements of essential minerals are well defined and supplied through least-cost formulations where an oversupply of nutrients is minimised. However, for many aquaculture species including *Penaeus monodon*, the mineral requirements are not well understood [1]. There are difficulties in assessing mineral requirements of *P. monodon* as they are reared in seawater, a medium which can provide a supply of over 20 minerals but the concentrations of which can be inconsistent with varying salinity. The bioavailability and utilisation of these water-borne minerals are not well established for this species.

The limited understanding of mineral requirements for *P. monodon* results in the aquaculture industry commonly including a mix of mineral sources at excessive rates to ensure requirements are met. An oversupply of dietary minerals can contribute to environmental pollution (i.e., particularly regarding phosphorus) which has negative consequences for the industry [2,3]. Refining our understanding of mineral requirements for *P. monodon* would, therefore, produce more efficient feeds and minimise unnecessary nutrient loss through effluent.

Mineral requirements for *P. monodon,* recommended by the National Research Council [1], reported nutrient requirement values for only three minerals: phosphorus (*P* = 0.7% diet), potassium (K = 1.2% diet) and copper (Cu = 10–30 ppm diet), as well as an ideal calcium (Ca):phosphorus (P) ratio of 1:1. The absences of recommendations for other minerals is concerning, as *P. monodon* represents 24% of global farmed prawn production and production is forecast to rise [4].

Minerals which have not been tested in *P. monodon* but have known necessity in other prawn species include macro-minerals: chloride (Cl), magnesium (Mg), sodium (Na) and sulphur (S); and trace minerals: cobalt (Co), iodine (I), manganese (Mn) and selenium (Se). A preliminary analysis of the mineral composition of adult black tiger prawn body (~8 g) reared in marine commercial-style ponds was conducted to provide an indication of which minerals may be of biological significance to this species. Two minerals of particular interest were strontium (Sr) and boron (B), which are present at 569 mg/kg and 9 mg/kg in whole prawns, respectively. Sr and B have demonstrated biological functions in other species [5,6,7,8] but have received limited focus in aquaculture nutrition.

The importance of a large array of minerals in diets for *P. monodon* can be assessed using a screening design, also known as a fractional factorial design. For example, Plackett-Burman (PB) [9] screening designs have been used recently to assess the importance of dietary factors in nutrition studies of production animals [10]. The PB statistical method analyses the main effects of factors on a response parameter, allowing 11 mineral factors to be assessed within 12 treatments. By employing a PB experimental design, a single mineral-factor can be assessed at two levels, in this case (i) with added mineral, +, and (ii) without added mineral, −, where six diets assess the mineral at each level. In the presence of mineral interactions, such a design is beneficial as the effect of each mineral-factor at level + or − is averaged over six diets, which contain different combinations of the other minerals to reduce the noise from mineral interactions. This would not be possible in a single factor deletion study as the difference in response of a single mineral-factor is determined by only two treatments where all other minerals are held constant except for the removal of one factor. Therefore, in a single deletion study, the effect of the mineral-factor may only be relevant in the presence of the same combination of minerals. A disadvantage of the PB design is that it assumes that not all mineral-factors will have a large effect on the response parameter. Therefore, a PB screening design with 12 treatments was applied to rank the importance of selected minerals to develop an initial assessment of mineral requirements.

The present study aimed to estimate the need for the following twelve minerals in diets for *P. monodon*: B, Ca plus P (considered in tandem at a 1:1 ratio), Co, Cu, Mg, Mn, K, Se, Na, Sr and Zn. The minerals were investigated as a two-level factor: (i) above expected mineral requirements by adding inorganic minerals, +, or (ii) below expected mineral levels as contributed only by the basal formulation, −. The allocation of each mineral level to diets was based on the PB screening design.

## 2. Materials and Methods 

### 2.1. Dietary Treatments

Twelve minerals (Ca and P were assessed in tandem) were investigated according to the PB design matrix (Table 1). It was not the intention of this study to investigate practical inclusion levels of minerals but rather to understand the ranked importance of a wide selection of minerals for prawn growth and nutrient assimilation into tissue. Thus, the inclusion rates of inorganic minerals added to the basal formulation were based on twice the levels reported in requirement studies or muscle composition concentrations when no requirement values have been determined for prawns. This was to ensure requirements for each mineral were met and to maximise the response in measured parameters. Furthermore, due to the variation in reported requirement values, studies were selected which most closely resembled the rearing conditions of this study, i.e., marine *Penaeid* prawns reared in high-salinity water.

Purified casein, gelatin and wheat starch-based diets were formulated to be iso-energetic and iso-nitrogenous and to contain inorganic sources of the target minerals in accordance with the PB design matrix where Diet 12 contained all inorganic mineral inclusions (Table 2). Diatomaceous earth was used as a dietary filler. Inorganic minerals were weighed in a fume hood and agitated in deionised water before being incorporated to the diet mix to prevent aeration of fine mineral particulates. Water was added, to approximately 30%, during mixing to form a dough which was subsequently screw-pressed (Dolly, La Monferrina, Castell’Alfero, Italy) through a 2 mm die and cut to pellet length of approximately 6 mm. Pellets were then steamed for 3 min and oven dried at 65 °C for 12 h.

A water stability assessment was performed on each diet in which ~1 g of feed was added to the aerated trial system (*n* = 4) without animals and recovered after 5 h by siphoning through a 200 µm screen. A 5 h time frame of water stability was selected to account for the length of time feed would be submersed in water during a feeding regime of five feedings per day. Recovered feed was dried in the oven at 105 °C to determine dry matter (DM) weight and a diet stability factor (%DM) was calculated using the formulae:

Diet water stability (%DM) after 5 h = 100 × (Feed recovered_g DM_/Feed offered_g DM_) (1)

### 2.2. Prawn Feeding Trial

A total of forty-eight (48) 100 L indoor experimental tanks were used to evaluate 12 treatments each with four replicate tanks. The number of tanks per treatment was determined using the online sample size calculator, Statulator^beta^ (©Statulator 2014, The University of Sydney, Australia). This program calculated that the minimum number of tank replicates required was 3.1, which was rounded up to 4. Specifically, the statistical power was set to 0.80 to calculate the minimal sample size [19]. Juvenile prawns were expected to gain 0.7 g/week and achieve a final weight gain (WG) of at least 6.2 g at the end of the feeding trial, based on previous feeding trials with 8 prawns/tank and 4 tank replicates/treatment, completed at the Bribie Island Research Centre, Australia. The typical standard deviation observed in previous juvenile prawn trials was 1.5 g/prawn for final weight. A 5% difference was used to calculate the effect size and thus, effect size of weight was 0.59 g/prawn.

Juvenile prawns (2.0 ± 0.29 g) were sorted and eight (8) prawns were allocated into each tank based on body weights so that all initial prawn weights were within one standard deviation. Samples of initial prawns (4 replicates of 8 prawns) were kept at −20 °C until analyses for nutrient retention calculations. Prawns were held in flow-through tanks with a single air diffuser for aeration and supplied with ozonated, UV-treated and -filtered sea water at a continuous rate of 0.6 L/min with water temperature, salinity and dissolved oxygen maintained at 29 °C, 38 g/L and 5.8 mg/L, respectively.

Prawns were housed for six weeks and fed by automatic feeders (06:00, 14:00, 18:00 and 20:00 h) and a single manual feed (10:00 h), totalling five feeds spread evenly throughout the day. Feed intake (FI) was monitored daily and rations were adjusted based on the presence of uneaten feed in tanks to ensure prawns were fed to satiation. The amount of feed delivered to each tank was recorded after the last ration was delivered. Uneaten feed was siphoned and collected into a 200 µm screen, rinsed with freshwater and pooled weekly per tank, before being oven dried (105 °C for 12 h) to determine FI. A 5 h water stability factor was used to adjust the amount of uneaten feed recovered, based on the time feed would be submerged in water and a corrected FI was calculated on a DM basis using the formula:

FI (g/prawn/day) = [Feed_in_ × DM − (Feed_out_/Diet water stability) × 100]/(7 × Number_prawn_) (2)

where Feed_in_ is the cumulative amount of feed as is delivered over 7 days, DM is the dry matter content of each feed, Feed_out_ is the amount of dry matter recovered from each tank after 7 days and Number_prawn_ is the number of live prawn.

### 2.3. Sample Collection and Analysis

Prawn mortalities and moults were collected and recorded daily. Mean body weights of prawns for each tank were determined on Days 21 and 42 from live individuals and feed efficiency (FE; gain:feed) was calculated. The diet effect on WG, FI and FE determined at Day 21 were reported in Appendix A and Appendix B. Prawns were fasted for 12 h prior to the conclusion of the trial (Day 42) to ensure emptied digestive tracts and minerals from digesta would not interfere with whole body analysis. Final weights of prawns were recorded and three prawns from each tank were euthanized by submersion in an ice slurry before being retained on ice for body composition analyses. Animal samples were stored frozen at −20 °C, before being freeze-dried using an Alpha 1–4 LD Plus. The diet and freeze-dried animal samples were then ground to pass a 1 mm mesh before analysis.

Samples of diets and whole prawns were analysed for total nitrogen (N) to determine crude protein (CP), gross energy (GE), crude lipid (CL), DM, ash content and mineral concentrations. All composition analyses were carried out in CSIRO laboratory facilities at the Queensland Bioscience Precinct, Queensland, Australia. DM was obtained gravimetrically after drying at 105 °C for 12 h. Ash content was obtained gravimetrically following combustion at 550 °C for 12 h in a muffle furnace. CP was calculated (N × 6.25) following determination of total N by combustion analysis using a Thermo Scientific Flash 2000 Elemental analyser. GE was determined by isoperibolic bomb calorimetry in a Parr 6200 oxygen bomb calorimeter with an 1108CL bomb for diets and whole prawn samples (Par Instrument Company, Moline, IL, USA). CL was extracted by using a modified Folch extraction [20] and determined gravimetrically. Diet and whole prawn mineral composition were analysed by Inductively Coupled Plasma–Mass Spectrometry (ICPMS) using an Elan DRCII ICMPS (Perkin Elmer Inc., Waltham, MA, USA) for multi-element determination of trace elements. Before ICPMS analysis, the samples were solubilised using microwave-assisted acid digestion (Milestone Srl, Sorisole, BG, Italy) following a modified Environmental Protection Agency 3051A method [21]. Two 20 mL samples of seawater were taken at the termination of the animal trial for ICPMS analysis of minerals.

Biomass gain, weight gain (WG), FE, moulting rate and nutrient/energy retention were calculated as follows:

Biomass gain (g) = Final weight of total prawns − Initial weight of total prawns(3)

WG (g)= W_2_ − W_1_ (4)

FE = (W_2_ − W_1_)/C (5)

Moulting rate (%/week) = 100 × [(Sum of (N_moults_week_/N_prawn_week_))/6 weeks] (6)

Nutrient retention (%) = 100 × [(Nutrient_%final prawn_ × W_2_ − Nutrient_%initial prawn_ × W_1_)/(Nutrient content_%feed_ × C)] (7)

where W_2_ and W_1_ are the final and initial wet body weight of the prawns, respectively, Feed_IN_ is the *as-is* weight of feed delivered, Feed_OUT_ is the DM weight of uneaten feed recovered, T is the duration in days, C is the total amount of feed consumed, N_moults_week_ is the amount of moults collected in a week and N_prawn_week_i_ is the number of prawns in the corresponding week.

### 2.4. Statistical Analysis

Experimental data of growth performance parameters derived from four replicate tanks were analysed in a one-way ANOVA to observe the variation between diets. ANOVA assumptions of normality of residuals and homogeneity of variances were tested using the Shapiro–Wilk and Levene tests, respectively. Multiple comparisons Tukey–Kramer test were obtained using statistical analysis software NCSS 11 (Kaysville, UT, USA). PB design analysis were completed using the ‘Fit Two Level Screening’ platform on JMP^®^, Version 14.0. (SAS Institute Inc., Cary, NC, USA) to obtain main effects of minerals across 12 treatments, using four replicates per treatment. Minerals were set as categorical values with two levels (‘−’ and ‘+’) to determine their relative importance on prawn response parameters. Coefficient estimates, t-ratios and probability levels for each mineral were reported. The main effects of the mineral at the two inclusion levels: ‘−’ and ‘+’, were calculated by averaging the response parameter of diets assigned with the corresponding level. The magnitude of the effect of each mineral to a parameter is explained by the coefficient estimate which is calculated by comparing the degree of change between the ‘+’ diets to the ‘−’ diets. The coefficient estimates are then used to rank minerals in terms of their influence. T-ratios describe the magnitude of the effect size for each mineral, whereby a negative value indicates a reduction in the response parameter and a positive value indicates an increase. A probability level of less than 5% was considered to be statistically significant.

### 2.5. Animal Research Ethics

Animal research ethics was not required for research using prawns as outlined by the CSIRO Queensland Animal Ethics Committee guidelines.

## 3. Results

### 3.1. Composition of Diets

Diet specifications and mineral concentrations of seawater are shown in Table 3. Analysed CP of diets ranged from 494 to 530 g/kg and GE ranged between 19.0 and 19.5 MJ/kg. Concentrations of minerals in diets 1–12 agreed with calculated concentrations of minerals allocated by the PB design where diets assigned with − had mineral concentrations that were below expected requirements and diets assigned with + achieved mineral concentrations that were above expected mineral requirements; with the exception of Zn. Thus apart from Zn, diets with low mineral assignment − were expected to be deficient in the respective mineral and conversely, diets with high mineral assignment + were expected to meet requirements of the respective mineral.

### 3.2. Prawn Culture

Survival across all treatments averaged 85% and prawns grew 5-fold in relation to their initial weight over the six-week period. One-way ANOVA of dietary treatments reported in Table 4 showed diet had a significant effect on biomass gain, FI, FE and survival at Day 42 and diet stability at 5 h. In particular, Diet 1 obtained inferior biomass gain, FE and survival compared to all other diets. The lowest diet stability of 61.4% and 63.2% was obtained by Diets 10 and 4, respectively, and this was significantly lower than the highest diet stability of 98.9% obtained by Diet 5.

Table 5, Table 6 and Table 7 show the results from the two-level screening analysis using the PB design. Only results that were statistically significant are listed in Table 5, Table 6 and Table 7 (complete results are provided in Appendix A, Appendix B, Appendix C and Appendix D including parameters measured at Day 21). Several minerals were significant for WG, FI, FE, moulting frequency, biomass gain, survival at Day 42 and diet stability as shown in Table 5. K and Se were the most influential minerals on WG based on coefficient estimates where additional K negatively influenced WG by 8.9% (7.67 vs. 8.42 g; *p* < 0.05) while additional Se had a positive influence on WG with an 8.6% increase (8.37 vs. 7.71 g; *p* < 0.05). Measurements of biomass gain and survival showed similar effects from additional minerals. Survival was negatively influenced by the inclusion of Ca + P and Zn while Mn improved survival (90.1 vs. 78.7%; *p* < 0.01). Similarly, biomass gain was negatively influenced by Ca + P inclusions (46.8 vs. 56.4 g; *p* < 0.05) and was improved with Mn (57.3 vs. 45.9 g; *p* < 0.05) and B (56.0 vs. 47.2 g; *p* < 0.05).

Day 42, Se addition had a negative trend on intake (0.438 vs. 0.446 g/prawn/day) and significantly improved FE (0.478 vs. 0.427; *p* < 0.01). Decreases in FI by Ca + P (0.417 vs. 0.467 g/prawn/day; *p* < 0.01), Mg (0.426 vs. 0.458 g/prawn/day; *p* < 0.05) and Mn (0.425 vs. 0.459 g/prawn/day; *p* < 0.05) inclusions were also coupled with improved FE at Day 42 (all *p*-values < 0.05). Positive trends in WG and FI from B addition resulted in a significant improvement in FE at Day 42 (0.472 vs. 0.433; *p* < 0.05).

Diet stability in seawater averaged 77.3% DM and ranged from 61.4% to 90.2%. The addition of K reduced diet stability by 23.5% (67.1% vs. 87.6%; *p* < 0.001).

### 3.3. Whole Prawn Body Composition

Table 6 outlines the influence of mineral inclusions on prawn body composition sampled at Day 42. Inclusion of Se increased body CL content by 9.7% (6.65 vs. 6.06%; *p* < 0.05) while Cu reduced lipid content by 13.4% (5.90 vs. 6.81%; *p* < 0.001). B inclusion reduced ash content by 9.2% (12.45 vs. 13.48%; *p* < 0.001). The effect of mineral inclusions on GE composition and CP composition was not significant (*p* < 0.05) and are provided in Appendix C.

Retention of major dietary macronutrients and energy, i.e., GE, CP, CL, and total minerals were influenced by mineral inclusion (Table 6; all mineral effects are provided in Appendix D). The inclusion of B and Mn significantly improved nutrient and energy retention. Boron improved GE, CP and CL retention (all *p* < 0.05). Manganese produced higher retentions of GE, CP and total metal retention (all *p* < 0.01). Seven out of the eleven mineral factors tested (Ca + P, K, Mn, Na, Se, Sr and Zn) influenced the retention of total minerals (all *p*-values < 0.05). 

Table 7 reports the effect of dietary mineral addition on body mineral concentrations. Concentrations of Co, Cu, K, Mg, Mn, P, Se and Zn in the body were significantly affected (*p* < 0.05) by dietary mineral additions. Minerals that were not significantly affected are listed in Appendix E. Dietary inclusion of Cu had the broadest influence on body mineral concentrations by significantly increasing Cu (37 to 104 mg/kg; *p* < 0.001) and Zn (39 to 44 mg/kg; *p* < 0.05) while reducing *p* (2941 to 2611 mg/kg; *p* < 0.05). Five out of the twelve minerals included in diets resulted in higher concentrations of the corresponding mineral in the body. These were Co, Cu, Mg, Mn and Se (all *p*-values < 0.05).

## 4. Discussion

### 4.1. Effect of Diets on Prawn Culture

The growth of *P. monodon* juvenile prawns over the 6-week period (>1.0 g/week) was comparable to other trials assessing minerals in purified diets [12,15,22,23,24]. Weight gain averaged 84% per week, which is satisfactory when compared to weight gains of *P. monodon* fed purified diets in other trials, Peñaflorida [12] (68%) and Lee and Shiau [25] (183%). The lower survival of prawns fed Diet 1 (62.8%) and Diet 2 (65.6%) was unexpected and could not be explained. It is noteworthy that Diets 1 and 2 both contained added Ca:P and Zn and did not have Mn. This result likely contributed to the survival response where Ca + P and Zn had a negative effect on survival while Mn had a positive effect on survival.

Mineral analysis of Diets 1–12 confirmed the formulated mineral content was achieved, except for Zn, and the mineral treatments had the desired effect of generating expected mineral deficiencies or adequacies from the absence or presence of inorganic minerals, respectively.

### 4.2. Effect of Minerals via a PB Screening Analysis 

Addition of trace minerals: B, Mn, Se and Zn generated significant improvements in growth and nutrient utilisation when analysed as a PB design. The biological importance of Mn, Se and Zn in other aquatic animals has been demonstrated [26]; receiving recent attention since the increased accessibility of organometallic products such as chelates with amino acids and yeast-encapsulated minerals for animal feeding. Briefly, Mn plays a significant role in muscle composition functioning as a cofactor in several enzyme systems [27], Se acts as an antioxidant as well as a catalyst for growth and hormone production [28] and Zn is a component of many metalloenzymes relating to carbohydrate and glucose catabolism [18]. These three minerals are found in insufficient quantities in seawater and so must be provided in the diet, which was evident from the present study as the inclusion of twice the dose rate had a significant positive influence on performance.

Dietary inclusion of minerals improved retention of CP, CL and GE in prawns and may underlie the observed improvements in growth. The trace minerals B, Cu, Mn and Se, as well as macro-mineral Mg, were the highest ranked minerals of importance in the screening analysis for macronutrient and energy retention. Mn and B were particularly important for improving the retention of nutrients and this finding is consistent with the expected functional role that Mn plays in metabolism of nutrients [27]. The necessity for B is far less established. B function in invertebrates is unknown but it has been suggested that at least two vertebrate phylogenetic classes require B for bone metabolism [7].

The majority of B research is focused in plants as this is the only group where B has been demonstrated as being essential [6]. As reported in the mineral concentrations of seawater, B is present at 25 mg/L and there has been evidence for dietary B requirement in marine animals under certain conditions. Responses to dietary B were most marked when an experimental animal (terrestrial and aquatic) was in the presence of a stressor that adversely altered hormonal or cellular membrane status such as cholecalciferol, Ca, Mg or K deprivation [29]. B supplementation stimulated growth during embryonic and early larval stages of rainbow trout and this response was more pronounced in the absence of Ca, Mg and Na salts in the incubation solutions [30]. In the present study, prawns may have been in various states of mineral deficiency, which may have contributed to the positive growth responses to B supplementation. The positive influence of B on nutrient retention is intriguing, and further study is warranted to identify the biological function of dietary B addition in crustaceans.

The effect of dietary supplementation of macro-minerals on prawn culture was not consistent across all parameters; however, based on FE, Ca + P and Mg addition were beneficial. In crustaceans, Ca is the major component of the exoskeleton while P and Mg are involved in the metabolism of fats, carbohydrates and proteins. Furthermore, it has been demonstrated across multiple crustacean species that Ca and P requirements should be investigated in tandem as an imbalance will disadvantage the utilisation of either mineral [31,32,33]. In *P. monodon*, a dietary Ca and P interaction was evident where Ca:P ratios of 1:1 achieved optimal mineral assimilation producing a 62% increase in WG and 89% recovery from soft-shelling [34] whereas P supplemented diets that were devoid or oversupplied with Ca deteriorated WG by 15.9% and 27.5%, respectively [12]. In the present study, dietary Ca and P ratio of 1:1 caused no increase in body P concentrations but improved total mineral retention and prawn FE. Based on these results, minerals which were not previously tested in *P. monodon*: B, Mg, Mn, Se, Zn, and previously tested minerals, Ca:P at a 1:1 ratio, were identified as important for prawn growth and nutrient utilisation. Thus, it will be instructive to confirm nutrient requirement values in a dose–response experiment.

### 4.3. Effects of Diet Stability and Mineral Bioavailability

Dietary K had a surprising negative effect on growth performance, most likely due to its negative impact on diet water stability. K was the only mineral to significantly reduce water stability, which it did by 30% (87.6 to 67.1%) after 5 h submersion. Dietary K was included at 2.4% which converted to a 4.6% inclusion rate of potassium chloride (KCl). KCl has a high solubility of 272.6 g/L in deionised water at 30 °C which is higher compared to sodium chloride (NaCl; 265.5 g/L) [35]. The high solubility and inclusion of KCl could lead to its loss and breakdown of the pellet as shown by the negative effect of K on diet stability. Furthermore, we observed that prawns fed the highest concentration of K had the lowest levels of this element in their tissues, the opposite of what was expected (see Appendix F). Low diet stability is associated with poor palatability, decline in intake and diminished nutritional value caused by leaching [36]. The reduced growth accompanying high dietary K may relate to the mineral solubilizing and the pellet losing its stability, more so than a toxic effect caused by the element. The use of a binder could minimize the negative effect on diet stability from the addition of highly soluble ingredients; however, the bioavailability of the mineral once ingested should be considered.

The chemical form of a mineral is critical to its bioavailability as it characterizes the likelihood for the element to form insoluble substances impeding digestion, or hydrolyze and increase nutrient leaching [37]. In this study, the response to a dietary mineral appeared linked to its assimilation in the prawn body. For example, addition of Co, Cu, Mg, Mn and Se resulted in higher concentrations in body and enhanced growth and/or nutrient retention. The benefits of higher mineral assimilation on prawn growth requires further investigation. Highly bioavailable sources such as chelated minerals have potential benefits in enhancing mineral assimilation in prawns as observed by Roy et al. [38] and Bharadwaj et al. [39]; however, any corresponding benefits to prawn growth or health have yet to be demonstrated.

### 4.4. Variation in Response to Minerals as Observed in This Study

The interim sampling point at Day 21 in this study showed some divergent results compared to the final sampling at Day 42. A greater number of minerals had an effect on WG and FE measured at Day 21, where there were 4 and 7 significant mineral factors, respectively, compared to Day 42, where there were only 2 and 5 significant mineral factors, respectively (Appendix A and Appendix C). Thus, minerals observed to be important for WG and FE after 3 weeks did not maintain a similar effect on WG and FE after 6 weeks. The results provide insights on the potential mineral uptake by prawns, where prawns fed mineral-deficient diets may be able to compensate through the consumption of moults and/or prawn cannibalism. Our laboratory data indicate moult casings were on average 40% of the entire mineral body balance of the moulting animal. Furthermore, moulting rates were 26.6%/week/prawn (i.e., one moult every 3.8 weeks), which is far lower than the expected levels to achieve the present 5-fold increase in body mass over 42 days [40]. Thus it is likely moult consumption by prawns occurred, despite our best attempts to remove casings and dead prawns daily. It highlights the need to manage the consumption of minerals from other sources in mineral requirement studies and the further insight provided by data collection at more than a single timepoint. Future studies should investigate the effect of exposure length to diets on mineral requirements of prawns.

## 5. Conclusions

The application of a PB screening design identified important dietary minerals for growth and nutrient retention parameters in juvenile *P. monodon*. This outcome may be a result of the intricate allocation which can assess a high number of factors in a reduced number of treatments [9]. This strategy, coupled with high inclusion levels of minerals, ensured that the dietary mineral effects were large enough to be detected by the two-level screening design. The most influential minerals to influence growth and nutrient utilization in juvenile *P. monodon* were B, Mg, Mn, Se, Zn and Ca + P as a 1:1 ratio. Defining the optimal inclusion levels of these minerals, should be the focus of future studies. Mineral solubility can dictate the utilization of a mineral source by influencing diet characteristics, e.g., diet stability. Therefore, the effect of mineral chemical form on diet stability and prawn bioavailability should be considered when completing mineral requirement research.

## Figures and Tables

**Table 1:** Mineral-supplement allocation for twelve treatments and reported mineral requirements or muscle composition derived from 9 studies. ‘−’, ‘+’ denotes the inclusion rate of ‘below expected requirements’ and ‘above expected requirements in diets’, respectively. Inclusion rates of mineral in ‘+’ diets were twice the amount of the ‘reported mineral requirement or muscle composition’ level.

Diet Number	PB Factor Allocation for 12 Treatments												Reported Mineral Requirements or Muscle Composition	Inclusion Rate of Mineral in ‘+’ Diets	Species and Material Used to Determine Requirement
	1	2	3	4	5	6	7	8	9	10	11	12			
B, mg/kg	−	−	+	−	−	−	+	+	+	−	+	+	8.7	17.4	*Penaeus platyceros* muscle [11]
Ca + P, g/kg	+	+	−	+	−	−	+	−	−	−	+	+	14	28	*Penaeus monodon* diets [12]
Co, mg/kg	−	+	−	−	−	+	+	+	−	+	−	+	1.2	2.4	Carnivorous prawns diets [13]
Cu, mg/kg	+	−	−	−	+	+	+	−	+	−	−	+	32	64	*Penaeus vannamei* diets [14]
K, g/kg	+	−	−	+	−	−	−	+	+	+	−	+	12	24	*Penaeus monodon* diets [15]
Mg, g/kg	−	+	−	−	+	−	−	−	+	+	+	+	3.5	7.0	*Litopenaeus vannamei* diets [16]
Mn, mg/kg	−	−	−	+	+	+	−	+	−	−	+	+	60	120	Carnivorous prawns diets [13]
Na, g/kg	+	−	+	−	−	+	−	−	−	+	+	+	10	20	Penaeid prawn diets [1]
Se, mg/kg	−	−	+	+	+	−	+	−	−	+	−	+	0.4	0.8	*Penaeu. vannamei* diets [17]
Sr, mg/kg	−	+	+	+	−	+	−	−	+	−	−	+	50	100	*Penaeus platyceros* muscle [11]
Zn, mg/kg	+	+	+	−	+	−	−	+	−	−	−	+	15	30	*Penaeus vannamei* diet [18]

**Table 2:** Dietary composition of casein and wheat starch-based diets for prawns.

Diet	1	2	3	4	5	6	7	8	9	10	11	12
Ingredient (%Diet)												
Base mixture ^1^	82.4	82.4	82.4	82.4	82.4	82.4	82.4	82.4	82.4	82.4	82.4	82.4
Diatomaceous earth	1.73	10.22	12.52	6.78	16.43	12.50	11.39	13.02	11.88	6.83	5.12	0.50
Sum of mineral premix	15.92	7.44	5.13	10.88	1.22	5.15	6.26	4.64	5.78	10.82	12.53	17.16
Mineral premix details ^2^												
H_3_BO_3_ (g/kg)			0.1				0.1	0.1	0.1		0.1	0.1
CoCl_2_ (mg/kg)		5				5	5	5		5		5
CuCl_2_ (g/kg)	0.1				0.1	0.1	0.1		0.1			0.1
MnSO_4_·H_2_O (g/kg)				0.37	0.37	0.37		0.37			0.37	0.37
Na_2_SeO_3_ (mg/kg)			4	4	4		4			4		4
C_4_H_6_O_4_Sr (g/kg)		0.24	0.24	0.24		0.24			0.24			0.24
ZnSO_4_·7H_2_O (g/kg)	0.13	0.13	0.13		0.13			0.13				0.13
Ca_3_(PO_4_)_2_ (g/kg)	36.1	36.1		36.1			36.1				36.1	36.1
NaPO_4_H_2_ (g/kg)	26.3	26.3		26.3			26.3				26.3	26.3
NaCl (g/kg)	50.8		50.8			50.8				50.8	50.8	50.8
MgO (g/kg)		11.6			11.6				11.6	11.6	11.6	11.6
KCl (g/kg)	45.8			45.8				45.8	45.8	45.8		45.8

^1^ Casein (37.4% diet), pregelatinised wheat starch (15.7%), gluten (7%), gelatin (8%), fishoil (6.0%), attractant (5%; as D-glucosamine, glycine, glutamic acid and alanine at 5:3:1:1), lecithin (1.0%), vitamin premix (1.0%; as Vitamin A, 2.5 MIU; vitamin D3, 1.25 MIU; vitamin E, 100 g; vitamin K3, 10 g; vitamin B1, 25 g; vitamin B2, 20 g; vitamin B3, 100 g; vitamin B5, 100 g; vitamin B6, 30 g; vitamin B9, 5 g; vitamin B12, 0.05 g; biotin, 1 g; vitamin C, 250 g; Banox E, 13 g), cholesterol (0.5%), TAU (0.4%), methionine (0.2%), astaxanthin (0.1%) and yttrium (0.1%). ^2^ Sigma-Aldrich, Castle Hill, Australia.

**Table 3:** Analyzed diet specifications and mineral concentrations of seawater.

**Diet**	**1**	**2**	**3**	**4**	**5**	**6**	**7**
Composition (g/kg DM)							
DM (% as is)	93	95	96	95	96	96	95
GE (MJ/kg)	19.1	19.0	19.4	19.1	19.0	19.1	19.2
CP	524	521	510	530	512	494	516
CL	54	55	63	56	60	60	64
Ash	206	216	218	208	218	218	218
Diet stability (%DM)	73	90	85	63	99	76	89
Diet pH	5.1	5.7	5.6	5.3	5.8	5.6	5.4
Minerals							
B (mg/kg)	0.6	3.1	15.9	0.6	2.0	0.1	15.9
Ca (g/kg)	14.7	13.1	4.9	12.5	4.9	5.3	12.5
Co (mg/kg)	0.3	2.1	0.3	0.3	0.3	2.4	2.3
Cu (mg/kg)	64.8	18.6	18.4	11.0	105.2	84.1	84.5
K (g/kg)	27.4	1.4	1.1	23.6	1.2	1.4	1.1
Mg (g/kg)	0.7	8.9	0.5	0.6	5.0	0.6	0.6
Mn (mg/kg)	8.8	11.9	5.6	124.9	128.0	121.4	9.7
Na (g/kg)	29.5	6.3	20.7	5.8	1.4	20.2	6.0
P (g/kg)	23.0	20.0	5.7	20.0	5.7	6.0	20.0
Se (mg/kg)	0.3	0.3	2.4	2.5	2.5	0.3	2.3
Sr (mg/kg)	12.8	100.8	94.3	97.6	5.8	90.8	11.6
Zn (mg/kg)	61.2	66.8	68.6	42.1	84.0	49.4	46.6
**Diet**	**8**	**9**	**10**	**11**	**12**	**Seawater Sample**	
Composition (g/kg DM)							
DM (% as is)	96	96	96	95	95		
GE (MJ/kg)	19.1	19.2	19.2	19.2	19.1		
CP	508	525	508	510	530		
CL	61	62	57	56	50		
Ash	222	223	220	211	211		
Diet stability (%DM)	74	67	61	86	65		
Diet pH	5.7	5.9	5.9	5.2	5.5		
Minerals							
B (mg/kg)	15.3	17.4	2.7	17.8	17.3	25 mg/L	
Ca (g/kg)	4.9	4.9	4.7	12.8	12.7	510 mg/L	
Co (mg/kg)	2.1	0.3	2.2	0.3	2.3	1 µg/L	
Cu (mg/kg)	15.7	73.2	11.4	9.5	63.9	3 µg/L	
K (g/kg)	22.3	24.0	23.8	1.3	24.6	2449 mg/L	
Mg (g/kg)	0.4	6.4	10.3	10.6	7.1	363 mg/L	
Mn (mg/kg)	118.6	6.9	7.0	124.9	125.8	18 µg/L	
Na (g/kg)	1.2	1.2	20.4	25.8	26.1	1516 mg/L	
P (g/kg)	5.1	5.8	5.6	20.8	21.0	389 µg/L	
Se (mg/kg)	0.2	0.2	2.4	0.2	2.9	51 µg/L	
Sr (mg/kg)	6.2	92.4	5.6	11.0	99.3	25 µg/L	
Zn (mg/kg)	64.6	43.5	39.5	38.6	57.1	9 µg/L	

**Table 4:** Effect of twelve dietary treatments on growth performance and survival of juvenile prawns at Day 42 and diet stability, as analyzed by a one-way ANOVA.

Diet	WG (g)	FI (g/Prawn/Day)	FE	Biomass Gain (g)	Survival (%)	Diet Stability (% DM After 5 h)
1	6.47	0.434 ^ab^	0.362 ^a^	25.6 ^a^	62.5 ^a^	72.4 ^ab^
2	8.50	0.459 ^ab^	0.460 ^abcd^	39.0 ^ab^	65.6 ^ab^	90.2 ^ab^
3	9.04	0.520 ^b^	0.431 ^abcd^	47.7 ^ab^	71.9^ab^	85.0 ^ab^
4	7.96	0.416 ^ab^	0.476 ^abcd^	47.8 ^ab^	81.3 ^ab^	63.2 ^a^
5	8.77	0.421 ^ab^	0.523 ^cd^	54.4 ^ab^	81.3 ^ab^	98.9 ^b^
6	7.40	0.489 ^ab^	0.374 ^a^	57.0 ^ab^	96.9 ^ab^	76.2 ^ab^
7	8.49	0.429 ^ab^	0.491 ^abcd^	52.1 ^ab^	81.3 ^ab^	89.3 ^ab^
8	8.57	0.457 ^ab^	0.457 ^abcd^	68.5 ^b^	100 ^b^	73.6 ^ab^
9	7.06	0.444 ^ab^	0.391 ^ab^	51.6 ^ab^	93.8 ^ab^	66.9 ^ab^
10	7.77	0.469 ^ab^	0.406 ^abc^	59.4 ^b^	96.9 ^ab^	61.4 ^a^
11	8.29	0.394 ^a^	0.518 ^bcd^	61.0 ^b^	93.7 ^ab^	86.1 ^ab^
12	8.20	0.371 ^a^	0.543 ^d^	55.0 ^ab^	87.5 ^ab^	64.9 ^ab^
SEM	0.537	0.025	0.026	6.382	7.065	7.045
*p*-value	0.062	0.012	<0.001	0.006	<0.001	0.006

^abcde^ Means within columns not sharing common suffixes are significantly different at the 5% level of probability, as determined by a multiple comparisons Tukey-Kramer test.

**Table 5:** Effect of significant mineral inclusions on growth performance parameters including WG (g) ^1^, FI (g/prawn/day) ^2^, FE (gain:feed) ^3^, moulting frequency (%/week) ^4^, biomass gain (g) ^5^, survival (%) ^6^ at Day 42 and diet stability (%DM after 5 h) ^7^ using a two-level screening analysis. *p*-values are denoted by an asterisk (* *p* < 0.05, ** *p* < 0.01, *** *p* < 0.001). t-ratios of minerals highlighted green have a positive effect and red have a negative effect on the performance parameter where the shading reflects the magnitude (i.e., darker shading has a higher magnitude of effect and vice versa).

Mineral	Performance Factor	Main Effect −	Main Effect +	Estimate	t-Ratio
B *	FE (gain:feed)	0.433	0.472	0.26	2.50
B *	Biomass gain (Tank weight gain; g)	47.2	56.0	8.80	2.40
Ca + P **	FI (g/prawn/day)	0.467	0.417	−0.05	−3.48
Ca + P **	FE (gain:feed)	0.430	0.475	0.31	2.97
Ca + P *	Moulting frequency (%/week)	21.1	32.1	10.9	2.60
Ca + P *	Biomass gain (Tank weight gain; g)	56.4	46.8	−9.60	−2.60
Ca + P *	Survival (%)	90.1	78.7	−11.50	−2.81
Cu *	Moulting frequency (%/week)	30.1	23.2	−11.7	−2.80
K *	WG (g)	8.4	7.7	−0.75	−2.41
K ***	Diet stability (%DM after 5 h)	87.6	67.1	−20.60	−5.10
Mn *	FI (g/prawn/day)	0.459	0.425	−0.03	−2.42
Mn ***	FE (gain:feed)	0.423	0.482	0.40	3.88
Mn *	Moulting frequency (%/week)	31.4	21.9	10.9	−2.30
Mn **	Biomass gain (Tank weight gain; g)	45.9	57.3	11.40	3.10
Mn **	Survival (%)	78.7	90.1	11.50	2.81
Mg *	FI (g/prawn/day)	0.458	0.426	−0.03	−2.20
Mg **	FE (gain:feed)	0.432	0.473	0.28	2.75
Se *	WG (g)	7.7	8.4	0.66	2.12
Se **	FE (gain:feed)	0.427	0.478	0.35	3.40
Zn **	Survival (%)	90.6	78.1	−12.50	−3.06

Ranked significant factors of importance according to coefficient estimates for each growth performance parameter: ^1^ K > Se. ^2^ Ca + P > Mn > Mg. ^3^ Mn > Se > Ca + P > Mg > B. ^4^ Cu > Ca + P > Mn. ^5^ Mn > Ca + P > B. ^6^ Zn > Ca + P = Mn. ^7^ Only significant factor was K.

**Table 6:** Effect of significant mineral inclusions on prawn body content (%) and retention (%) sampled at Day 42 including ash composition ^1^, CL composition ^2^, GE retention ^3^, CP retention ^4^, CL retention ^5^, and total mineral retention ^6^ using two-level screening analysis. *p*-values are denoted by an asterisk (* *p* < 0.05, ** *p* < 0.01, *** *p* < 0.001). t-ratios of minerals highlighted green have a positive effect and red have a negative effect on the performance parameter where the shading reflects the magnitude (i.e., darker shading has a higher magnitude of effect and vice versa).

Mineral	Performance Factor	Main Effect −	Main Effect +	Estimate	t-Ratio
B ***	Ash composition (%body)	13.48	12.45	−1.02	−4.01
B *	GE retention (MJ/kg)	12	13.6	1.56	2.44
B *	CP retention (%body)	17.6	19.6	2.01	2.32
B *	CL retention (%body)	13.7	15.7	2.04	2.13
Ca + P ***	Total mineral retention (%body)	7.22	4.24	−2.98	−5.77
Cu ***	CL composition (%body)	6.81	5.9	−0.91	−4.08
Cu *	CL retention (%body)	15.7	13.7	−2.05	−2.14
K ***	Total mineral retention (%body)	7.02	4.44	−2.57	−4.98
Mn **	GE retention (MJ/kg)	11.7	13.9	2.2	3.45
Mn **	CP retention (%body)	17.2	20.1	2.95	3.4
Mn **	CL retention (%body)	13.3	16.1	2.83	2.94
Mn **	Total mineral retention (%)	4.77	6.69	1.92	3.72
Mg **	CL retention (%body)	13.1	16.2	3.12	3.25
Na ***	Total mineral retention (%body)	6.97	4.5	−2.47	−4.78
Se *	CL composition (%body)	6.06	6.65	0.59	2.67
Se *	CL retention (%body)	13.5	15.8	2.29	2.38
Se *	Total mineral retention (%body)	5.06	6.41	1.34	2.6
Sr **	Total mineral retention (%body)	6.5	4.97	−1.53	−2.97
Zn *	Total mineral retention (%body)	5.14	6.33	1.19	2.31

Ranked significant factors of importance according to estimates for each growth performance parameter: ^1^ Only significant factor was B. ^2^ Cu > Se. ^3^ Mn > B. ^4^ Mn > B. ^5^ Mg > Mn > Se > Cu > B. ^6^ Ca + P > K > Na > Mn > Sr > Se > Zn.

**Table 7:** Effect of significant mineral inclusions on mineral content of prawn body sampled at Day 42 using two-level screening analysis. *p*-values are denoted by an asterisk (* *p* < 0.05, ** *p* < 0.01, *** *p* < 0.001). t-ratios of minerals highlighted green have a positive effect and red have a negative effect on the performance parameter where the shading reflects the magnitude (i.e., darker shading has a higher magnitude of effect and vice versa).

Mineral	Performance Factor	Main Effect −	Main Effect +	Estimate	t-Ratio
B **	Cu body content (mg/Kg)	65	76	10.93	2.8
Co **	Co body content (mg/Kg)	0.10	0.16	0.06	3.09
Cu ***	Cu body content (mg/Kg)	37	104	67.12	17.16
Cu *	P body content (mg/Kg)	2941	2611	−330.94	−2.07
Cu *	Zn body content (mg/Kg)	39	44	4.69	2.47
Mg *	Mg body content (mg/Kg)	926	1008	81.44	2.36
Mn *	Cu body content (mg/Kg)	75	66	−9.54	−2.44
Mn ***	Mn body content (mg/Kg)	1.32	5.48	4.16	11.42
Na *	K body content (mg/Kg)	4110	4456	345.95	2.24
Se ***	Se body content (mg/Kg)	0.74	1.24	0.50	6.68

## Abbreviations

CP	crude protein
CL	crude lipid
DM	dry matter
FE	feed efficiency
FI	feed intake
GE	gross energy
PB	Plackett-Burman
N	total nitrogen
WG	weight gain

## Appendix A

**Table A1:** Effect of twelve dietary minerals at two inclusion levels (−/+) on weight gain (g) at Day 21 and 42, moulting frequency (g/week), biomass gain (g) and survival (%) at Day 42 using two-level screening analysis. Factors are sorted by |estimates| and significant factors are marked by an asterisk (* *p* < 0.05, ** *p* < 0.01, *** *p* < 0.001).

**Factor**	**Main Effect −**	**Main Effect +**	**Estimate**	**t-Ratio**
Weight gain (Day 21, g)				
K **	4.02	3.57	−0.45	−2.95
Zn **	3.58	4.02	0.44	2.90
Mn *	3.60	4.00	0.40	2.61
Se *	3.61	3.99	0.38	2.51
Cu	3.95	3.65	−0.30	−2.00
Co	3.69	3.91	0.22	1.45
Na	3.91	3.69	−0.22	−1.45
Ca + P	3.75	3.85	0.10	0.63
B	3.76	3.83	0.07	0.48
Mg	3.83	3.76	−0.07	−0.46
Sr	3.76	3.83	0.07	0.45
**Factor**	**Main Effect** **−**	**Main Effect +**	**Estimate**	**t-Ratio**
Weight gain (Day 42, g)				
K *	8.42	7.67	−0.75	−2.41
Se *	7.71	8.37	0.66	2.12
Cu	8.35	7.73	−0.62	−2.00
B	7.81	8.28	0.47	1.50
Zn	7.83	8.26	0.43	1.39
Na	8.23	7.86	−0.36	−1.17
Mn	7.89	8.20	0.31	1.00
Co	7.93	8.16	0.22	0.72
Ca + P	8.10	7.98	−0.12	−0.38
Mg	7.99	8.10	0.11	0.35
Sr	8.06	8.03	−0.03	−0.11
**Factor**	**Main Effect** **−**	**Main Effect +**	**Estimate**	**t-Ratio**
Moult frequency (% per week)				
Cu **	30.1	23.2	−11.7	−2.8
Ca + P *	21.2	32.1	10.9	2.6
Mn *	31.4	21.9	−9.5	−2.3
Co	30.1	23.2	−6.9	−1.7
Mg	29.8	23.4	−6.4	−1.6
K	29.2	24.1	−5.1	−1.2
B	28.0	25.3	−2.7	−0.6
Zn	25.5	27.8	2.3	0.6
Na	26.9	26.3	−0.6	−0.2
Se	26.3	26.9	0.6	0.2
Sr	26.8	26.5	−0.3	−0.1
**Factor**	**Main Effect** **−**	**Main Effect +**	**Estimate**	**t-Ratio**
Biomass gain (Tank weight gain; g)				
Mn **	45.9	57.3	11.4	3.1
Ca + P *	56.4	46.8	−9.6	−2.6
B *	47.2	56.0	8.8	2.4
Co	48.0	55.2	7.2	2.0
Zn	54.8	48.4	−6.4	−1.8
Cu	53.9	49.3	−4.6	−1.3
Sr	53.5	49.7	−3.8	−1.0
Mg	49.8	53.4	3.6	1.0
Se	50.5	52.7	2.3	0.6
Na	52.2	51.0	−1.3	−0.4
K	51.9	51.3	−0.5	−0.1
**Factor**	**Main Effect −**	**Main Effect +**	**Estimate**	**t-Ratio**
Survival (Day 42; %)				
Zn **	80.7	88.0	12.5	3.1
Mn **	80.7	88.0	−11.5	−2.8
Ca + P **	84.9	83.9	11.5	2.8
B	78.7	90.1	−7.3	−1.8
Co	85.4	83.3	−7.3	−1.8
K	85.9	82.8	−5.2	−1.3
Mg	90.6	78.1	−4.2	−1.0
Sr	90.1	78.7	3.1	0.8
Se	83.9	84.9	2.1	0.5
Cu	82.3	86.5	1.0	0.3
Na	81.8	87.0	−1.0	−0.3

## Appendix B

**Table A2:** Effect of twelve dietary minerals at two inclusion levels (−/+) on diet stability (% DM after 5 h), feed intake (g/prawn/day) at day 21 and 42 and feed efficiency (FE) at Day 21 and 42 over the 6-week trial period using two-level screening analysis. Factors are sorted by |estimates| and significant factors are marked by an asterisk (* *p* < 0.05, ** *p* < 0.01, *** *p* < 0.001).

**Factor**	**Main Effect −**	**Main Effect +**	**Estimate**	**t-Ratio**
Diet stability (% DM after 5 h)				
K ***	87.6	67.1	−20.6	−5.1
Zn	73.8	80.9	7.0	1.7
Na	80.4	74.3	−6.0	−1.5
Sr	80.3	74.4	−5.9	−1.5
Co	78.8	75.9	−2.8	−0.7
Cu	76.6	78.1	1.5	0.4
Mg	76.6	78.1	1.5	0.4
Ca + P	77.0	77.7	0.7	0.2
B	77.1	77.7	0.6	0.2
Se	77.6	77.1	−0.5	−0.1
Mn	77.5	77.2	−0.4	−0.1
**Factor**	**Main Effect −**	**Main Effect +**	**Estimate**	**t-Ratio**
Feed intake (Day 21; g/prawn/day)				
Mg *	0.372	0.341	−0.03	−2.70
Ca + P *	0.371	0.342	−0.03	−2.54
Cu	0.367	0.346	−0.02	−1.81
Mn	0.365	0.348	−0.02	−1.52
Se	0.364	0.349	−0.01	−1.29
Co	0.350	0.363	0.01	1.20
Na	0.350	0.363	0.01	1.16
B	0.360	0.353	−0.01	−0.63
Sr	0.353	0.360	0.01	0.55
K	0.359	0.354	−0.01	−0.46
Zn	0.354	0.358	0.00	0.34
**Factor**	**Main Effect −**	**Main Effect +**	**Estimate**	**t-Ratio**
Feed intake (Day 42; g/prawn/day)				
Ca + P **	0.467	0.417	−0.05	−3.48
Mn *	0.459	0.425	−0.03	−2.42
Mg *	0.458	0.426	−0.03	−2.20
Cu	0.453	0.431	−0.02	−1.48
K	0.452	0.432	−0.02	−1.39
Sr	0.434	0.45	0.02	1.10
B	0.448	0.436	−0.01	−0.85
Se	0.446	0.438	−0.01	−0.60
Na	0.438	0.446	0.01	0.59
Co	0.438	0.446	0.01	0.51
Zn	0.44	0.444	0.00	0.24
**Factor**	**Main Effect −**	**Main Effect +**	**Estimate**	**t-Ratio**
FE (Day 21; gain:feed)				
Mn ***	0.471	0.553	0.45	5.34
Se ***	0.474	0.550	0.41	4.95
Zn ***	0.483	0.540	0.31	3.70
K **	0.539	0.485	−0.29	−3.51
Ca + P **	0.485	0.538	0.29	3.42
Na **	0.535	0.489	−0.25	−2.97
Mg *	0.494	0.529	0.19	2.28
B	0.502	0.522	0.11	1.26
Co	0.506	0.517	0.06	0.71
Cu	0.515	0.508	−0.04	−0.46
Sr	0.512	0.512	0.00	−0.03
**Factor**	**Main Effect** **−**	**Main Effect +**	**Estimate**	**t-Ratio**
FE (Day 42; gain:feed)				
Mn ***	0.423	0.482	0.40	3.88
Se **	0.427	0.478	0.35	3.40
Ca + P **	0.430	0.475	0.30	2.97
Mg **	0.432	0.473	0.28	2.75
B *	0.433	0.472	0.26	2.53
Na	0.466	0.439	−0.19	−1.82
K	0.466	0.439	−0.18	−1.79
Zn	0.443	0.463	0.14	1.33
Sr	0.459	0.446	−0.09	−0.91
Cu	0.458	0.447	−0.07	−0.72
Co	0.450	0.455	0.03	0.32

## Appendix C

**Table A3:** Effect of twelve dietary minerals at two inclusion levels (−/+) on CL, ash, CP and GE content of prawn bodyes using two-level screening analysis. Factors are sorted by |estimates| and significant factors are marked by an asterisk (* *p* < 0.05, ** *p* < 0.01, *** *p* < 0.001).

**Factor**	**Main Effect** **−**	**Main Effect +**	**Estimate**	**t-Ratio**
CL (% body)				
Cu ***	6.81	5.90	−0.91	−4.08
Se *	6.06	6.65	0.59	2.67
Zn	6.57	6.14	−0.42	−1.90
Ca + P	6.55	6.16	−0.39	−1.76
Mg	6.17	6.54	0.38	1.70
B	6.18	6.53	0.35	1.58
Na	6.23	6.47	0.24	1.08
Co	6.27	6.44	0.17	0.75
K	6.42	6.29	−0.12	−0.55
Mn	6.39	6.32	−0.08	−0.34
Sr	6.32	6.39	0.07	0.30
**Factor**	**Main Effect** **−**	**Main Effect +**	**Estimate**	**t-Ratio**
Ash (% body)				
B ***	13.48	12.45	−1.02	−4.01
Sr	12.75	13.18	0.43	1.68
Mn	12.77	13.16	0.39	1.55
Se	13.11	12.82	−0.29	−1.14
Co	13.09	12.84	−0.24	−0.95
Cu	13.08	12.85	−0.24	−0.92
Mg	12.86	13.07	0.21	0.81
K	12.88	13.05	0.18	0.69
Zn	13.04	12.89	−0.14	−0.56
Ca + P	12.94	12.99	0.06	0.22
Na	12.98	12.95	−0.03	−0.13
**Factor**	**Main Effect** **−**	**Main Effect +**	**Estimate**	**t-Ratio**
CP (% body)				
Ca + P	72.6	74.9	2.23	3.03
B	74.5	73.0	−1.42	−1.93
Mn	74.3	73.2	−1.09	−1.48
Cu	73.2	74.3	1.09	1.47
Se	74.2	73.3	−0.84	−1.15
Sr	73.4	74.1	0.63	0.86
K	74.0	73.5	−0.51	−0.69
Zn	73.6	73.9	0.32	0.44
Mg	73.7	73.8	0.18	0.24
Co	73.7	73.8	0.11	0.15
Na	73.7	73.8	0.03	0.04
**Factor**	**Main Effect** **−**	**Main Effect +**	**Estimate**	**t-Ratio**
GE (MJ/kg body)				
Se	18.5	18.8	0.32	1.72
Sr	18.6	18.8	0.18	0.99
K	18.8	18.6	−0.16	−0.86
Co	18.6	18.7	0.12	0.64
Cu	18.7	18.6	−0.08	−0.46
Mn	18.7	18.7	0.03	0.15
Zn	18.7	18.7	0.03	0.14
Na	18.7	18.7	−0.03	−0.14
B	18.7	18.7	0.02	0.09
Mg	18.7	18.7	0.01	0.07
Ca + P	18.7	18.7	0.00	0.01

## Appendix D

**Table A4:** Effect of twelve dietary minerals at two inclusion levels (−/+) on GE retention, CP retention, CL retention, ash retention and total mineral retention of prawn body using two-level screening analysis. Factors are sorted by |estimates| and significant factors are marked by an asterisk (* *p* < 0.05, ** *p* < 0.01, *** *p* < 0.001).

**Factor**	**Main Effect** **−**	**Main Effect +**	**Estimate**	**t-Ratio**
GE retention (MJ/kg)				
Mn **	11.7	13.9	2.20	3.45
B *	12.0	13.6	1.56	2.44
Mg	12.2	13.4	1.18	1.85
Se	12.2	13.4	1.18	1.85
Ca + P	12.4	13.2	0.74	1.15
Co	12.5	13.2	0.71	1.11
Sr	13.1	12.5	−0.56	−0.88
Na	13.0	12.6	−0.41	−0.65
K	12.9	12.7	−0.18	−0.29
Cu	12.9	12.7	−0.18	−0.28
Zn	12.8	14.4	−0.01	−0.02
**Factor**	**Main Effect** **−**	**Main Effect +**	**Estimate**	**t-Ratio**
CP retention (%)				
Mn **	17.2	20.1	2.95	3.40
B *	17.6	19.6	2.01	2.32
Mg	17.8	19.4	1.61	1.86
Ca + P	18.1	19.2	1.16	1.34
Se	18.1	19.2	1.12	1.30
Co	18.1	19.2	1.09	1.26
Sr	19.1	18.1	−0.98	−1.13
K	19.0	18.3	−0.69	−0.80
Na	18.8	18.5	−0.29	−0.33
Zn	18.8	18.5	−0.27	−0.32
Cu	18.7	18.5	−0.19	−0.22
**Factor**	**Main Effect** **−**	**Main Effect +**	**Estimate**	**t-Ratio**
CL retention (%)				
Mg **	13.1	16.2	3.12	3.25
Mn **	13.3	16.1	2.83	2.94
Se *	13.5	15.8	2.29	2.38
B *	13.7	15.7	2.04	2.13
Cu *	15.7	13.7	−2.05	−2.14
Ca + P	14.0	15.3	1.28	1.33
Co	14.1	15.3	1.21	1.26
Na	14.2	15.2	1.04	1.08
Zn	14.9	14.4	−0.51	−0.53
K	14.4	14.9	0.47	0.49
Sr	14.8	14.5	−0.31	−0.32
**Factor**	**Main Effect** **−**	**Main Effect +**	**Estimate**	**t-Ratio**
Total mineral retention (%)				
Ca + P ***	7.22	4.24	−2.98	−5.77
K ***	7.02	4.44	−2.57	−4.98
Na ***	6.97	4.50	−2.47	−4.78
Mn **	4.77	6.69	1.92	3.72
Sr **	6.50	4.97	−1.53	−2.97
Se *	5.06	6.41	1.34	2.60
Zn *	5.14	6.33	1.19	2.31
Co	6.17	5.30	−0.88	−1.70
Cu	5.30	6.16	0.86	1.67
Mg	5.43	6.04	0.61	1.19
B	5.83	5.63	−0.20	−0.38

## Appendix E

**Table A5:** Effect of dietary mineral inclusions on prawn body mineral concentrations (mg/Kg) using two-level screening analysis. PB statistical values for each mineral is outlined. t-ratios with asterisks (*, **, ***) denote significant *p*-values defined as * *p* < 0.05, ** *p* < 0.01, *** *p* < 0.001.

Mineral Parameters	Prawn Body Mineral Concentrations (t-Ratio)											
	B mg/Kg	Ca mg/Kg	Co mg/Kg	Cu mg/Kg	K mg/Kg	Mg mg/Kg	Mn mg/Kg	Na mg/Kg	P mg/Kg	Se mg/Kg	Sr mg/Kg	Zn mg/Kg
B	−0.29	−0.82	1.36	2.8 **	−0.21	−1.15	1.25	−1.77	0.89	1.28	0.48	1.91
Ca + P	0.43	0.56	−1.71	−0.66	0.35	0.5	−1.37	0.78	1.77	1.87	0.82	−0.88
Co	0.29	−0.56	3.09 **	0.7	−1.34	0.5	1.14	−0.85	−1.75	1.95	−0.85	−0.05
Cu	0.42	−1.15	0.27	17.16 ***	−1.22	−1.94	−0.87	−1.53	−2.07 *	−0.64	0.16	2.47 **
K	0.38	0.97	1.62	−0.61	−0.32	0.41	1.94	−0.79	0.27	−0.47	1.38	0.64
Mg	−0.39	1.95	0.4	−1.19	1.03	2.36 *	−1.46	1.21	0.47	0.91	−0.35	−0.52
Mn	−0.41	0.5	−0.54	−2.44	0.3	0.35	11.42 ***	0.04	0.63	−1.52	−0.7	−0.76
Na	0.13	0.57	0.66	−1.9	2.24 *	0.86	−1.15	1.98	1.93	−1.52	0.14	0.01
Se	−0.39	−0.14	0.02	−0.35	−0.15	−0.37	−0.91	−0.41	0.19	6.68 ***	−0.78	−0.79
Sr	−0.03	−1.54	0.71	0.51	−1.2	−1.27	0	−0.14	−0.39	−1.63	−1.48	−1.46
Zn	−0.67	−1.27	−0.68	−0.57	−1.7	−1.81	0.07	−0.96	−1.05	−1.9	−0.84	−1.43

## Appendix F

**Table A6:** Effect of dietary mineral inclusions on prawn body mineral retention (%) using two-level screening analysis. PB statistical values for each mineral is outlined. t-ratios with asterisks (*, **, ***) denote significant *p*-values defined as * *p* < 0.05, ** *p* < 0.01, *** *p* < 0.001.

Mineral Parameters	Prawn Body Mineral Retention (t-Ratio)											
	B (%)	Ca (%)	Co (%)	Cu (%)	K (%)	Mg (%)	Mn (%)	Na (%)	P (%)	Se (%)	Sr (%)	Zn (%)
B	−12.52 ***	0.44	1.37	3.33 **	2.74 **	3.66 ***	3.47 **	3.31 **	1.87	4.42 ***	−0.19	4.07 ***
Ca + P	−7.31 ***	−11.24 ***	−0.15	1.69	0.90	−2.64 *	−4.25 ***	−10.63 ***	−13.54 ***	0.93	−3.31 **	1.94
Co	7.07 ***	0.59	−4.83 ***	−0.42	−2.56 *	2.81 ***	−1.31	−3.37 **	0.28	−0.78	1.28	0.37
Cu	9.05 ***	−1.11	−0.37	−7.58 ***	−0.27	−3.87 ***	−0.22	2.96 **	−2.51 *	−3.78 ***	−1.87	0.09
K	−7.21 ***	1.07	0.67	1.46	−20.60 ***	1.28	2.00	3.39 **	0.85	−0.52	0.74	1.30
Mg	−11.03 ***	2.60 *	0.53	0.67	0.97	−26.85 ***	0.63	3.35 **	0.47	2.19 *	1.57	1.86
Mn	9.53 ***	1.85	−0.12	2.69 *	1.33	4.26 ***	−8.17 ***	4.38 ***	1.41	2.45 *	1.87	1.45
Na	8.84 ***	−1.07	0.01	0.74	0.45	−5.13 ***	0.11	−14.74 ***	−0.31	−0.92	−1.22	0.77
Se	−8.73 ***	1.41	−0.53	−0.52	1.40	0.89	1.39	−3.02 **	0.94	−9.12 ***	0.95	0.02
Sr	9.18 ***	−2.72 **	0.68	−1.77	−3.37 **	−0.72	0.93	−3.99 ***	−1.80	−3.05 **	−10.60 ***	−2.17 *
Zn	−9.33 ***	−0.21	−1.05	−1.81	−1.21	0.38	−2.30 *	3.72 ***	0.71	−1.09	0.49	−7.23 ***
